# The Role of Long Non-coding RNAs in the Pathogenesis of RA, SLE, and SS

**DOI:** 10.3389/fmed.2018.00193

**Published:** 2018-07-03

**Authors:** Yunzhen Gao, Shasha Li, Zhongjian Zhang, Xinhua Yu, Junfeng Zheng

**Affiliations:** ^1^Institute of Psychiatry and Neuroscience, Xinxiang Medical University, XinXiang, China; ^2^Priority Area Asthma and Allergy, Research Center Borstel, Airway Research Center North, Members of the German Center for Lung Research, Borstel, Germany

**Keywords:** rheumatoid diseases, long non-coding RNAs, rheumatoid arthritis, systemic lupus erythematosus (SLE), Sjögren's syndrome

## Abstract

Rheumatoid diseases are a group of systemic autoimmune diseases which affect multiple organs with largely unknown etiology. In the past decade, long non-coding RNAs (lncRNAs) have emerged as important regulators of biological processes and contribute deeply to immune cell development and immune responses. Substantial evidences have been accumulated showing that LncRNAs involved in the pathogenesis of the rheumatoid diseases, including rheumatoid arthritis (RA), systemic lupus erythematosus (SLE) and Sjögren's syndrome (SS). In this review, we summarize literature combined with bioinformatics methods to analyze the unique and common lncRNAs patterns in rheumatoid diseases and try to reveal the important function of lncRNAs in RA, SLE and SS.

## Introduction

Autoimmune diseases are disorders caused by the break of the immune tolerance to autoantigens which is marked by emerging of autoreactive T cells and autoantibodies. Autoimmune diseases can be categorized into two groups based on organ-specificity. One group is organ-specific autoimmune disease that affects particular organs, and the other group is systemic autoimmune diseases that affect multiple organs (1). Rheumatoid diseases are a group of systemic autoimmune diseases, including rheumatoid arthritis (RA), systemic lupus erythematosus (SLE), Sjogren's syndrome (SS), polymyositis and dermatomyositis etc. (1). Rheumatoid diseases often share some common clinical and immunological features. For example, SS is closely related to other two rheumatoid diseases, RA and SLE (2). SS develop alone as primary SS (pSS), while it is referred to as secondary Sjogren's syndrome(2ndSS) when it occurs with other autoimmune diseases. The 2ndSS always coexist with SLE and RA, wherein 9–19% SLE patients (3, 4) and 4–31% RA patients (5, 6) develop the 2ndSS. In terms of immunological features, RA, SLE, and SS are all characterized with anti-nuclear antibodies (ANA) and rheumatoid factor (RF) (7). In addition, anti-Ro/ SSA and anti-La/SSB autoantibodies which are diagnostic markers for SS also can be detected in both SLE and RA, although with relatively low prevalence (8). Besides clinical and immunological features, the three rheumatoid diseases also share some common features at the molecule level. Analyses of gene expression profiles of peripheral blood mononuclear cell (PBMC) demonstrate that RA, SLE and SS share some common gene expression profiles and biological processes (9, 10). Finally, the three rheumatoid diseases also share some susceptibility genes, such as PTPN22 (11), STAT4 (12) and IRF5 (13–15). Taken together, the common features among RA, SLE, and SS in clinical, immunological and molecule levels suggest that they share some causes in their pathogenesis.

Long non-coding RNAs (lncRNAs) are molecules longer than 200 nucleotides in length (16). LncRNAs are RNAs with little or no protein-coding capacity (17). Since the first lncRNA discovered in the early 1990s (18), around 172,216 transcripts have been annotated in the human according to the NONCODE database (http://www.noncode.org/analysis.php) (17). As small RNA sponging and molecular scaffolds, lncRNAs play an important role in regulation of gene expression through multiple mechanisms (19–22). lncRNAs are actively involved in the regulation of many biological processes, including development and function of immune cells. Recently, a body of evidence has been accumulated showing that lncRNAs are involved in the pathogenesis of rheumatoid diseases, such as SS, SLE and RA (23–25).

In this review, we aim to provide some scientific insights into the role of lncRNAs in rheumatoid diseases. We summarize the recent progresses and discuss the role of LncRNAs in the pathogenesis of RA, SLE, and SS, and highlight those lncRNAs associated with multiple rheumatoid diseases. In addition, by comparing rheumatoid disease-associated proteins retrieved from Coremine Medical (http://www.coremine.com/medical/#search) with proteins regulated by lncRNA associated with rheumatoid diseases, we try to explore the mechanism behind the role of lncRNAs in RA, SLE, and SS.

## Long non-coding RNAs function and mechanisms

According to the criterion defined by GENCODE (26), lncRNAs are subclassified into antisense lncRNA, sense lncRNA, bidirectional lncRNA, and long intergenic noncoding RNAs (lincRNA) (27). Since the discovery of the first lncRNA (18), our knowledge about lncNRAs has been considerably increased. LncRNAs interact with RNA or DNA through complementary base-paring, and interact with protein via direct interaction (28, 29). Interactions between lncRNA with RNA, DNA, and proteins enable lncRNAs to regulate gene expression at multiple levels, including transcription, posttranscription, translation, post-translation, and epigenetic modification.

### Transcriptional regulation

LncRNAs can mediate both cis- and trans-regulation of transcription. Acting as enhancer associated RNAs (e-RNAs) and affect neighboring intra-chromosomal genes in cis-regulation manner (30). E-RNAs are transcribed from enhancer regions harboring specific chromatin states such as H3K4me1 and H3K27ac (31). E-RNAs can regulate neighboring genes expression through recruiting transcription factors or complexes and guiding them to the specific region of target genes (32). Also, lncRNAs can interact with the transcription factors to form the preinitiation complex and thus either activate or inhibit gene transcription (33, 34). Another group of lncRNAs called ncRNA-activating are able to recruit the transcriptional co-activator mediator to promote chromatin looping of neighboring genes, resulting in the transcriptional activation of those genes (35). Finally, the process of transcription of lncRNA is also capable to affect adjacent genes transcription (36).

Apart from cis-regulation, lncRNAs also regulate genes expression in trans-acting manner. For example, lncRNA 7SK negatively regulates transcription elongation factor P-TEFb complex by interacting with high mobility group protein A1 (HMGA1) (37, 38) and lncRNA B2 acts as brakes which hold back the advancement of RNA polymerase II (39, 40).

### Post-transcriptional regulation

LncRNAs play a role in post-transcriptional regulation by influence the pre-mRNA procession and mRNA stability (36). Alternative splicing of pre-mRNA is an important event in the eukaryotes gene transcription, which provides diverse transcripts. For example, interaction between nuclear enriched abundant transcript 1 (NEAT1) and serine/arginine-rich splicing factor 5 (SRp40) influences the function of splicing factors that act on splicing of the PPARγ2 gene during adipogenesis (41). Metastasis associated lung adenocarcinoma transcript 1 (MALAT1, also known as NEAT2) is another lncRNA contributing to the pre-mRNA splicing. Previous studies suggest that the interaction between MALAT1 and serine/arginine splicing factors act a pivotal part in cancer progression (42), synapse formation (43), and growth signal responsive genes expression (44).

LncRNAs also regulate mRNA stability via multiple ways. First, lncRNAs can act as a sponge to absorb microRNA and thus regulate the stability of target mRNA (45). For example, Linc-MD1 affects skeletal muscle differentiation and disease though sponging miR-133 and miR135 (46). In addition, lncRNAs are also able to regulate mRNA stability by interacting with the 3′UTR or the coding region of target mRNAs. A good example is half-STAU1-binding site RNAs (1/2-sbsRNAs), a group of lncRNAs which form imperfect base-pairs with an Alu element in the 3′ UTR of a target mRNA and another Alu element in a 1/2-sbsRNAs, and this formation is required for the STAU1-mediated messenger RNA decay (45). Another example is lncRNA maternally expression gene 3 (MEG3) which recruit polypyrimidine tract-binding protein 1 (PTBP1) to bind to the coding region of small heterodimer partner (SHP) mRNA and subsequently decay the mRNA (47). Interestingly, in a feedback-regulatory fashion, SHP inhibits cAMP response element-binding protein (CREB) mediated expression of MEG3 (47), suggesting a crosstalk between lncRNA and mRNA.

### Translational regulation

By interacting with the apparatus of translation, lncRNAs enhance or suppress gene expression at the translational level. For example, Ubiquitin carboxy-terminal hydrolase L1 antisense RNA 1 (UCHL1-AS1) interacts with polysomes and thus promotes the translation of UCHL1 mRNA (48). And non-coding RNA activated by DNA damage (NORAD) maintains genomic stability by sequestering PUMILIO protein, which repress the stability and translation of the target mRNA (49). By interacting with translation initiation factor 4E (eIF4E), growth arrest-specific transcript 5 (GAS5) inhibit the translation of c-Myc mRNA (50). Finally, LncMyod, a lncRNA encoded next to the Myod gene directly binds to IGF2-mRNA-binding protein 2 (IMP2) and negatively regulates IMP2-mediated gene translation such as c-Myc (51).

### Post-translational regulation

Post-translational modification of protein refers to the process of adding or removing chemical components on protein, as well as the process of protein folding and degradation (52). Phosphorylation and acetylation of a protein usually is associated with its activation status, while ubiquitination of a protein means protein degradation. LncRNAs are involved in the regulation of post-translational modification of proteins at multiple levels. For example, lncPRESS1, a p53-regulated transcript, interacts physically with SIRT6 and prevents SIRT6-mediated histone H3K56 and H3K9 deacetylation, therefore maintaining the pluripotency of human embryonic stem cells (53). Another example is lncRNAs associated with breast cancer brain metastases (lnc-BM), which binds to JH2 domain of JAK2 protein thus mediate oncostatin-M and signal transducer and activator of transcription 3 (STAT3) phosphorylation by increasing JAK2 kinase activity (54). Furthermore, lnc-DC, a lncRNA exclusively expressed in human conventional DCs, regulates the phosphorylation of STAT3 by direct binding to STAT3 and prevent its dephosphorylation by SHP1 (55).

Besides function in abovementioned transcriptional, post-transcriptional, translational and post-translation levels, lncRNAs regulate gene expression via some other mechanisms. LncRNAs can affect epigenetic regulation by altering the DNA methylation (56), histone modifications (57) and genetic imprinting (58). In addition, some lncRNAs containing short open reading frames are translated into functional micro-peptides which might carry their function in the regulation of gene expression (59). Taken together, lncRNAs regulates biological processes interacts by interacting with other molecules such as miRNA, DNA, and protein, which is an important mechanism for maintaining life function.

## Long non-coding RNA in rheumatoid disease

Given the important role of lncRNAs in regulating gene expression, it is conceivable to speculate that lncRNAs participates in various physiological and pathological processes of rheumatoid diseases. Using a variety of methods, such as microarray, quantitative reverse transcriptase-polymerase chain reaction (RT-PCR) and whole transcriptome sequencing, the researchers demonstrated that many lncRNAs are differentially expressed in rheumatoid patients and healthy controls, and this differential expression is associated with disease characteristics. These results suggest that lncRNAs play a role in developing of Rheumatoid disease (60). Therefore, exploration of the function of lncRNAs will help us clarify the mechanism of rheumatoid disease and provide new diagnostic markers and therapeutic targets.

### LncRNAs in rheumatoid arthritis

The pathogenesis of RA is complex and involves the interaction of host factors (e.g., genetic susceptibility and sex hormones) with environmental players (e.g., bacterial or viral infection) (61). So far, 27 lncRNAs have been implicated to play a role in RA (Table 1).

**Table 1:** Long non-coding RNA implicated in Rheumatoid arthritis.

**LncRNAs**	**Sample**	**Chromosome locus (hg38)**	**Expression**	**Related proteins**	**References**
HOTAIR	PBMC, exosome	Chr12: 53962308–53974956	Up-regulated	MMP2, MMP3	(62)
ENST00000483588	RAFLSs	Chr17: 16428987–16470648	Up-regulated	ATAD3A,NDUFA4L2	(63)
ENST00000438399	RAFLSs	Chr10: 29698475–29713107	Down-regulated	PTPRQ,PTHLH	(63)
uc004afb.1	RAFLSs	Chr: 41007011–41074625	Down-regulated	TNFRSF11B	(63)
ENST00000412143	RAFLSs	Chr: 31173735–31177899	Down-regulated		(63)
ENST00000452247	RAFLSs	Chr10: 52296848–52314128	Down-regulated	ZNF154,WISP3	(63)
LOC100652951	T cells		Up-regulated		(64)
LOC100506036	T cells	Chr2: 96811821–96815889	Up-regulated	SMPD1, NFAT1	(64)
ENST00000445339	PBMC	Chr1: 230002730–230007162	Down-regulated		(65)
ENST00000506982	PBMC	Chr4: 143953634–144126359	UP-regulated		(65)
MALAT1	RAFLSs	Chr11: 65497679–65504494	UP-regulated	Caspase3, Caspas9	(66)
RNA143598	Serum		UP-regulated		(25)
RNA143596	Serum		UP-regulated		(25)
HIX0032090	Serum		UP-regulated		(25)
GHCgamma1	Serum		UP-regulated		(25)
XLOC_002730	Serum		UP-regulated		(25)
ZFAS1	Synovial, RAFLSs	Chr20: 49278177–49289260	UP-regulated	miR27a	(67)
lincRNA-p21	Whole blood	Chr17: 29057473–29078961	Down-regulated	REAL,NF-KB	(68)
H19	synovial tissue	Chr11: 1995682–2001470	UP-regulated		(69)
NR_024118	Mouse model			SOCS3,MMP1,MMP3	(70)
C5T1	PBMC	Chr9: 120942252–120952831	UP-regulated	C5	(71)
GAS5	CD4 T cells, B cells	Chr1: 161035166–161038537	Down-regulated	mTOR,GR	(72)
ENST00000456270	PBMC	Chr7:117604790–117647415	Up-regulated		(73)
NR_002838	PBMC	Chr18:41480270–41520597	Up-regulated		(73)
NR_026812	PBMC	Chr21:35037935–35039426	Down-regulated		(73)
uc001zwf.1	PBMC	Chr15:48426827–48428972	Down-regulated		(73)

The first lncRNA implicated in RA is H19. In 2003, Stuhlmueller et al. reported that H19 expression was significantly higher in the synovial tissues (ST) from patients with RA and osteoarthritis (OA) than that in normal control or individual with joint trauma (69). In the cultured synovial fibroblasts (SFBs), the H19 expression is low but can be strongly induced by serum starvation. Interestingly, this starvation-induced increase of H19 expression in RA-SFBs is significantly higher than in OA-SFBs and control SFBs (69). Significant over expression of H19 RNA and its increased sensitivity to starvation/cytokine regulation in RA implicate a role of H19 in the pathogenesis of RA.

Since upregulation of ZFAS1 has been observed in cancers and promotes cell migration and invasion (74–76), Ye et al. investigated the role of this lncRNA in RA. They found that ZFAS1 expression level was increased in synovial tissue from patients with RA compared with that in controls (67). Their study further proved that lncRNA ZFAS1 promoted the migration and invasion of RA FLS is a miR-27a-dependent manner (67).

Another lncRNA associated in RA is growth arrest-specific 5 (GAS5) which acts as a potent repressor of the glucocorticoid receptor (GR) through its RNA “glucocorticoid response element (GRE)” (77). In 2016, Mayama and colleagues examined GAS5 levels in multiple immune related diseases (72). They found the expression level of GAS5 was significantly reduced in the CD4+ T cells and B cells from patients with RA compared with that in controls, Mayama et al. (72).

LncRNA C5T1 is located in a genomic region comprising a RA risk locus TRAF1-C5 (71). C5T1 expression is positively correlated with C5 mRNA, and knockdown of C5T1 specifically decreases the level of C5 mRNA, suggesting that C5T1 positively regulates C5 expression (71). Since C5 is overexpressed in inflamed joint of patients with RA (78) and C5-deficient mice are resistant to the development of RA model (79), it is conceivable that C5T1, as the C5 gene regulator, is associated with RA.

T cells play important roles in the pathogenesis of RA (80). To investigate the role of lncRNAs in RA patients, Lu et al. determine 10 potentials aberrantly expressed lncRNAs in T cells from 39 RA patients and 17 health cohort by RT-PCR (64). Among the 10 candidate lncRNAs, LOC100652951 and LOC100506036 are up-regulated. Interestingly, treated with tumor necrosis factor (TNF) antagonists could decrease LOC100652951 expression level. In addition, knockdown of LOC100506036 by siRNA could inhibit the production of the interferon gamma and decrease the expression level of nuclear factor of activated T cells (NFAT) and sphingomyelin phosphodiesterase 1 (SMPD1) (80). Their results indicate that LOC100652951 may be involved in the production of cytokines and LOC100506036 may contribute to the inflammatory response in RA.

Compared with detecting candidate lncRNAs, determination of the expression profile of lncRNAs by microarray, and subsequent validation with quantitative RT-PCR is a high throughput strategy for identification of disease-related lncRNAs. In 2015, Song et al. analyzed the expression profile of 83 lncRNAs in PBMC and blood exosome from 28 RA patients and 10 controls (62). Compared with healthy controls, the expression level of HOTAIR is increased in both PBMC and blood exosome of RA patients. Functional study shows that HOTAIR contributes to the migration of activated macrophage as well as the MMP2 and MMP13 activation (62). This indicates that HOTAIR might involve in the inflammation and the dissolution of bone and cartilage matrix in RA (62).

Using a biochip capable of detecting 40,173 lncRNAs, Yuan et al. analyzed the expression profiles of lncRNA in three pairs of samples (73). They identified 2,099 lncRNAs which were differentially expressed in PBMC between RA and controls. With PBMC from 36 RA patients and 24 healthy controls, the authors verified four significantly differentially expressed lncRNAs. ENST00000456270 and NR_002838 were up-regulated, whereas NR_026812 and uc001zwf.1 were down-regulated in patients with RA as compared to controls (73). Furthermore, the expression level of ENST00000456270 strongly correlates with the level of IL-6 and TNF-α in serum and the simplified Disease Activity Index (SDAI) of the RA patients (73).

Another study determining the expression profile of lncRNA in PMBC from RA patient was performed by a Chinese research group. Through a microarray capable of detecting 30,586 human lncRNA, Luo and colleagues determined the expression profile of lncRNAs in PBMC from 10 patients with RA and 10 well-matched health controls (65). The results revealed that 139 lncRNAs were significantly differentially expressed between RA patients and controls. The authors further validated the two most significantly deregulated lncRNAs, ENST00000445339 and ENST00000506982 using quantitative RT-PCR in the 24 patients with RA and 24 controls.

Fibroblast-like synovial cells (FLSs), one of the key effector cells in RA synovium, have attracted increasing attention (81). To explore lncRNAs expression pattern in the FLSs of RA, Zhang et al. determined the of lncRNAs profiles in FLSs of 10 RA patients and 10 patients with trauma as controls (63). Among the 30,586 lncRNAs detected by the microarray, 135 were differentially expressed in FLS between RA group and controls. By quantitative RT-PCR, the authors confirmed four differentially expressed lncRNAs, including ENST00000483588 which was up-regulated in RA FLSs as well as ENST00000438399, uc004afb.1, and ENST00000452247 which were down-regulated in RA FLSs as compared with controls. Notably, the expression level of ENST00000483588 is positively correlated with C-reactive protein (CRP) level and the simplified Disease Activity Index (SDAI) score (*r* = 0.79, *P* < 0.01). In addition, four lncRNAs show good diagnostic value for RA with the area under ORC curve ranging from 0.85 to 0.97 (63).

Besides cells, serum samples also be used for identification of RA-related lncRNAs. To identify lncRNAs associated with RA, Xu et al. investigated the expression profile of lncRNAs in serum samples from 3 RA patients and 3 health controls by microarray and then verified the interested lncRNAs in 43 RA patients and 40 healthy controls by quantitative RT-PCR (25). This study has identified 5 significantly up-regulated lncRNAs in RA as compared with controls, including RNA143598, RNA143596, HIX0032090, IGHCgamma1 and XLOC_002730. Moreover, these lncRNAs are positively correlated with some immunological and clinical features of RA, including rheumatoid factor (RF), erythrocyte sedimentation rate (ESR), anti-cyclic citrullinated peptide (anti-CCP) antibody and disease course (25).

Besides lncRNAs which are differentially expressed and functionally related with disease pathogenesis, some other lncRNAs have been implicated in RA because they are associated with therapeutic efficacy in the disease. Quercetin, a free oxygen radical scavenger (82), is effective in the management of RA (83). *In vitro*, Quercetin decreases the viability and promotes the apoptosis of FLS from patients with RA (84). Interestingly, treated with quercetin induces the expression of lncRNAs MALAT1 and knockdown of MALAT1 decreases the expression of caspase-3 and caspase-9 thus inhibits the FLS apoptosis induced by quercetin. Therefore, this study suggests that MALAT1 is involved in the quercetin-induced apoptosis of FLS and thus MALAT1 maybe have a therapeutic efficacy in RA. Zicao (purple gromwell) is a traditional Chinese herbal medicine, its major active component is Shikonin (66). Shikonin possesses anti-inflammatory property and can effective reduce the incidence and severity of RA in a collagen-induced arthritis mouse model. These results suggest that Shikonin is a good candidate of RA protective medicine (85). In the CIA model, Shikonin treatment increases the expression of lncRNAs NR_024118 in the joint of diseased mice through increasing the acetylation of H3 in the promoter of the lncRNAs (70). The knockdown of NR_024118 could reverse the effects of shikonin on proinflammatory cytokines and MMPs, suggesting that shikonin exerts it therapeutic effect in mouse model of RA via lncRNA-NR024118. Another lncRNA implicated in the therapeutic efficacy in RA is LincRNA-p21, one of lncRNAs induced in p53-mediated DNA damage response (68). Methotrexate (MTX) is an anchor therapy for the management of RA. In RA patients, MTX treatment increases the expression of lincRNA-p21 and decreases the level of p65 (RelA) phosphorylation compared with untreated RA patients (86), Interestingly, the basal levels of lincRNA-p21RA is reduced, while basal levels of RelA increased in RA patients (86). In addition, T cells from RA patients have deficiencies in DNA damage response as indicated by upregulation of DNA-PKcs (87). This suggests that MTX treatment might act through upregulating the lincRNA-p21 expression and thus prevent DNA damage and apoptosis in T cells. This note is supported by *in vitro* finding that MTX treatment induces the expression level of lincRNA-p21 through a DNA-PKcs-dependent mechanism in primary T cells or Jurkat cells (86).

### LncRNAs in systemic lupus erythematosus

SLE Patient usually presents highly heterogeneous in pathogenesis and disease features, which makes it difficult to understand the etiology of SLE. In recent years, emerging evidence has demonstrated that lncRNAs are involved in the pathogenesis of SLE, which brings new insights into SLE research (Table 2).

**Table 2:** Long non-coding RNA implicated in systemic lupus erythematosus.

**LncRNAs**	**Sample**	**Chromosome locus (hg38)**	**Expression**	**Related proteins**	**References**
GAS5	CD4 T cells, B cells, mouse model, plasma	Chr1:161035166-161038537	Down-regulated	mTOR,GR	(72, 88, 89)
NEAT1	PBMC	Chr11: 65422798-65445540	UP-regulated	IL-6, CXCL10	(90)
Linc0949	PBMC		Down-regulated		(91)
Linc0597	PBMC, plasma		Down-regulated		(89, 91)
Lnc-DC	plasma	Chr17: 60083566-60088467	Down-regulated		(89)
MALAT-1	PBMC	Chr11: 65497679-65504494	UP-regulated	IL-21	(92)
uc001ykl.1	PBMC		Down-regulated	CD46, TRAF1, LEPR, CXCR4, PTN, LEPR, CCR6, SUMO4, STAT1, RECK, DK6	(93)
ENST00000448942	PBMC	Chr6: 137854703-137868233	Down-regulated	MYD88,CD46,TRAF1,CXCR4,NFE2L2,LEPR,TAP1,IKZF1,STAT1,TNFSF10,TRIM69,RECK,TNFSF10	(93)
RP11-875O11.1	PBMC		Up-regulated		(94)
CTC-471J1.2	PBMC	Chr19: 52058100-52095757	Down-regulated		(94)

As in RA, the expression level of GAS5 is also decreased in the patients with SLE compared to control (72), indicating that GAS5 is associated with SLE. The association between GAS5 and SLE has been further supported by some other evidence. In BXSB mice, a spontaneous mouse model for SLE, GAS5 gene is located within a disease-susceptibility interval and carries multiple polymorphism which may account for the disease-related gene expression profile, suggesting that it is a candidate susceptibility gene in this SLE model (88). Furthermore, a recent study performed by Wu et al. demonstrated that the plasma level of GAS5 in SLE patients is significantly lower than that in healthy controls (89). Besides GAS5, Wu et al. also identified two other SLE-associated lncRNAs, lnc-DC and linc0597. Lin-DC, an intergenic lncRNAs, is exclusively expressed in human conventional DCs (55). Lnc-DC bound directly to STAT3 in the cytoplasm and promote the activation of STAT3. Knockdown of lnc-DC impaired DC differentiation *in vitro* and *in vivo* and reduced capacity of DCs to stimulate T cell activation (55). Compared with controls, the expression level of lnc-DC is significantly decreased in the plasma from patients with SLE (89). Furthermore, compared with SLE without nephritis, the lnc-DC expression level is significantly increased in lupus nephritis, making lnc-DC a promising marker distinguishing the two subgroups of SLE. In contrast to GAS5 and lnc-DC, the expression of linc0597 is significantly increased in plasma of SLE patients as compared with controls (89).

The association between linc0597 and SLE is confirmed by another study in which Wu et al. examined the expression levels of 4 immune-related lncRNAs in PBMC from 102 SLE patients and 76 healthy controls (91). They found that the expression levels of linc0949 and linc0597 were significantly decreased in SLE patients compared with those in controls. Furthermore, correlation analysis has demonstrated that linc0949 is negatively correlated with disease activity and positively correlated with complement component C3. In addition, the level of linc0949 is negatively associated with lupus nephritis and cumulative organ damage (91).

LncRNAs MALAT1 is associated with RA (84) and involved in the development and metastasis of cancer (95). To explore the role of MALAT1 in the pathogenesis of SLE, Yang et al. analyzed the expression of MALAT1 in PBMC from 39 SLE patients and 45 matched normal controls (92). They found that MALAT1 was abnormally increased in the patients with SLE and predominantly expressed in monocytes. In monocytes of patients with SLE, silencing MALAT1 significantly reduced the expression of IL-21 (92), an important cytokine in the pathogenesis of SLE. Furthermore, this study has also demonstrated that MALAT-1 exerts its detrimental effects by regulating silent information regulator 1 (SIRT1) signaling. Nuclear enriched abundant transcript 1 (NEAT1), a lncRNA often colocalized with MALAT1, has also been implicated in SLE. In 2016, Zhang et al. detected the level of NEAT1 in the PBMC from 39 SLE patients and 50 normal controls (90). They found that the NEAT1 level was significantly increased in SLE patients and the expression level of NEAT1 was positively correlated with disease activity of SLE (90). In both human monocyte cell line and primary monocytes, LPS or pam3cks4 stimulation could increase the expression of NEAT1. In addition, silencing of NEAT1 significantly reduced the expression of a group of chemokines and cytokines such as IL-6, CXCL10, etc., mainly through affection the late MAPK pathways, especially the phosphorylation of JNK and ERK.

High-throughput method such as microarray also apply to identify disease associated lncRNAs in SLE. In 2017, Li et al. analyzed the expression profiles of lncRNAs in T cells from SLE patients and healthy controls (93). Using quantitative RT-PCR, the authors verified that two lncRNAs uc001yk1.1 and ENST00000448942 were significantly downregulated in SLE patients with compared to controls. Moreover, the expression level of ENST00000448942 is correlated with anti-Sm antibodies and ESR (erythrocyte sedimentation rate), whereas the expression level of uc0011yk1.1 is correlated with ESR and C-reactive protein (93). Another study determining expression profile of lncRNAs was performed by Luo et al. through PBMC from SLE patients and controls (94). The results indicated that 8,868 lncRNAs (3,657 upregulated and 5,211 downregulated) were differentially expressed in SLE samples compared with the control group (94). By quantitative RT-PCR, the authors verified the upregulation of RP11-875O11.1 and the down-regulation of CTC-471J1.2 (94).

### LncRNAs in sjögren's syndrome

SS is a systemic autoimmune disease featured by dysfunction of exocrine glands (96). So far, 11 lncRNAs have been suggested to be associated with SS (Table 3).

**Table 3:** Long non-coding RNA implicated in primary Sjögren syndrome.

**LncRNAs**	**Sample**	**Chromosome locus (hg38)**	**Expression**	**Related proteins**	**References**
ENST00000420219	labial salivary glands	Chr13: 30357741-30377145	Up-regulated	CD6, AGARP2, LILRB1, RLTPR	(97)
ENST00000455309	labial salivary glands	Chr2: 111489103-111510997	Up-regulated	ARHGAP30, CCR5, GIMAP4, HIST1H2AI, ICAM1, PRKCQ, ZNF831, GNGT2, KIF20B, LILRB1	(97)
ENST00000546086	labial salivary glands	Chr12: 68333252-68442216	Up-regulated	LAPTM5, CFP, CXCR4, HCST, SUSD3	(97)
LINC00426	labial salivary glands	Chr13: 30340265-330373914	Up-regulated		(97)
LINC02384	labial salivary glands	Chr12: 68431845-68451696	Up-regulated		(97)
Lnc-UTS2D-1:1	labial salivary glands	Chr3: 191213290-191234605	Up-regulated		(97)
n336161	labial salivary glands	Chr14: 22392040-22392553	Up-regulated	AGAP2, GNGT2, ICAM1, LILRB1, RLTPR, ARHGAP30	(97)
n340599	labial salivary glands	Chr5: 119268907-119393381	Up-regulated	ACY3, CD19, CYTH4, RABGAP1L, RAC2, TLR9, TRAF1, WDFY4	(97)
NR_002712	labial salivary glands	Chr2: 218059155-218061290	Up-regulated		(97)
TCONS_l2_00014794	labial salivary glands	Chr2: 111195866-111495161	Up-regulated	CCL20, CFP, CXCR4, HCST, LEF1, SUSD3	(97)
TMEVPG1	PBMC	Chr12: 67989444-68021327	Up-regulated	INF-g, HLA-DRB, HLA-DOB, SSA, ESR, IgG, Ets-1, T-bet	(98)

LncRNA TMEVPG1 [also named Ifng-AS1 and Nettoie Salmonella pas Theiler's (NeST)] is located on the DNA stands opposite to interferon gamma (*IFNG*) coding gene and it can promote the transcription of *IFNG* as an enhancer (99). TMEVPG1 is expressed predominately in T cells (CD4 and CD8) and NK cells (100). In 2016, Wang and colleagues reported that the expression level of TMEVPG1 is increased in CD4^+^ T cells in SS patients compared with that in controls (98). Moreover, the expression of TMEVPG1 is positively correlated with levels of anti-SSA antibody, erythrocyte sedimentation rate (ESR), total IgG amount and the proportion of Th1 cells. In addition, *in vitro* study showed that the knockdown of TMEVPG1 could decrease the proportion of Th1 cells in CD4^+^ T cells from patients (98).

To identify whether the dysfunction of lncRNAs is involved in the pathogenesis of pSS, Shi et al. analyzed the expression profile of lncRNAs in labial salivary glands of patients with pSS and controls by microarray (97). Using a chip with a total capacity of 63,431 lncRNAs, They found 1243 lncRNAs were differentially expressed in pSS patients compared with controls, including 890 upregulated and 353 downregulated lncRNAs. By quantitative RT-PCR, the authors validated eight upregulated lncRNAs, named ENST00000420219.1, ENST00000455309.1, n336161, NR_002712, ENST00000546086.1, Lnc-UTS2D-1:1, n340599 and TCONS_l2_00014794 (97). The authors further analyzed the correlation between those eight lncRNAs and the clinical characteristics of pSS. The results revealed strong correlations between these lncRNAs and pSS characteristics, including β2 microglobulin, erythrocyte sedimentation rate (ESR), rheumatoid factor (RF), amount of IgA and IgM, visual analog scale (VAS) of parotid swelling and VAS of dry eyes (97).

### A role of LncRNAs in RA, SLE, and SS

As mentioned in the introduction, RA, SLE, and SS share many common clinical features at immunological and molecular levels (60). However, the molecular mechanisms behind the common features among rheumatoid diseases are not well understood. Association between lncRNAs and rheumatoid diseases might shed some new light on our understanding the pathogenesis of rheumatoid diseases.

**Figure 1:**
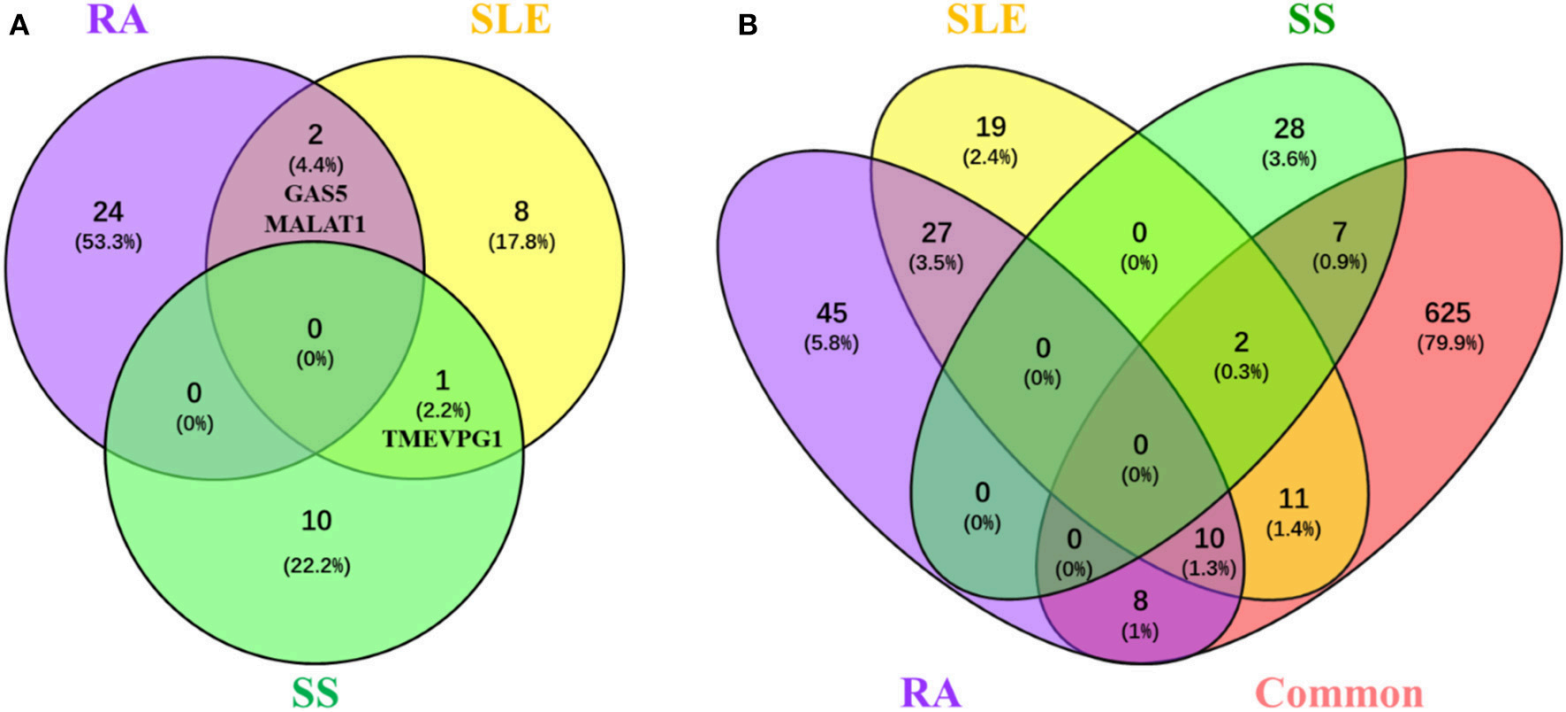
LncRNAs and their related proteins inplicated in RA, SLE, and pSS. **(A)** Venn diagram of the lncRNA implicated in RA, SLE, and pSS. Lists of lncRNAs that implicated in RA, SLE, and SS, respectively, were submitted to Venny webset (http://bioinfogp.cnb.csic.es/tools/venny/index.html) to analyze lncRNAs shared by multiple diseases. The number and percentage of lncRNAs in each fraction are depicted in the corresponding fraction. LncRNAs implicated in at least two diseases are depicted. RA, Rheumatoid arthritis; SLE, systemic lupus erythematosus; SS, Sjogren syndrome. **(B)** Venn diagram of the proteins interacted with lncRNAs that are implicated in RA, SLE, and SS. Using lncRInter websver (http://bioinfo.life.hust.edu.cn/lncRInter/), we obtained the proteins related with lncRNAs. For those lncRNAs, did not record in the lncRInter, we got its related proteins by searching literature manually. We also obtain 663 proteins which common to pathological basic of RA, SLE, and pSS by searching coremine database (http://coremine.com/medical/#search). The list of proteins interacted with lncRNAs that are implicated in RA, SLE, and pSS, respectively, and the 663 common proteins were submitted to Venny webset (http://bioinfogp.cnb.csic.es/tools/venny/index. html) to analyze lncRNA-related proteins shared by multiple diseases. The number and percentages of proteins in each fraction are depicted in the corresponding fraction. LncRNA-related proteins associated with at least two diseases are depicted. RA, Rheumatoid arthritis; SLE, systemic lupus erythematosus; SS, Sjogren syndrome.

Among the tens of lncRNAs involved in RA, SLE and SS, those associated with multiple diseases are special interesting since lncRNAs involved in the pathogenesis of multiple diseases would provide a hint for interpreting common features of rheumatoid diseases. Although lncRNAs implicated in RA, SLE, or SS, none of them is shared in all three rheumatoid diseases. However, one lncRNA, TMEVPG1, has been implicated in SLE and SS, and two lncRNAs, MALAT1 and GAS5, have been implicated in RA and SLE (Figure 1A).

It is conceivable that lncRNAs affect the development of rheumatoid disease via regulating the expression disease related proteins. To explore the possible mechanisms of lncRNA and related proteins in rheumatoid diseases, we collected 663 proteins have been suggested involved in the common pathological progress of RA, SLE and SS by either retrieving them from the coremine database (http://coremine.com/medical/#search) or manually from literature. Notably, 18 out of 90 proteins related to lncRNAs implicated in RA, 23 out of 69 proteins related to lncRNAs implicated in SLE and 9 out of 37 proteins related to lncRNAs implicated in SS (Figure 1B).

**Figure 2:**
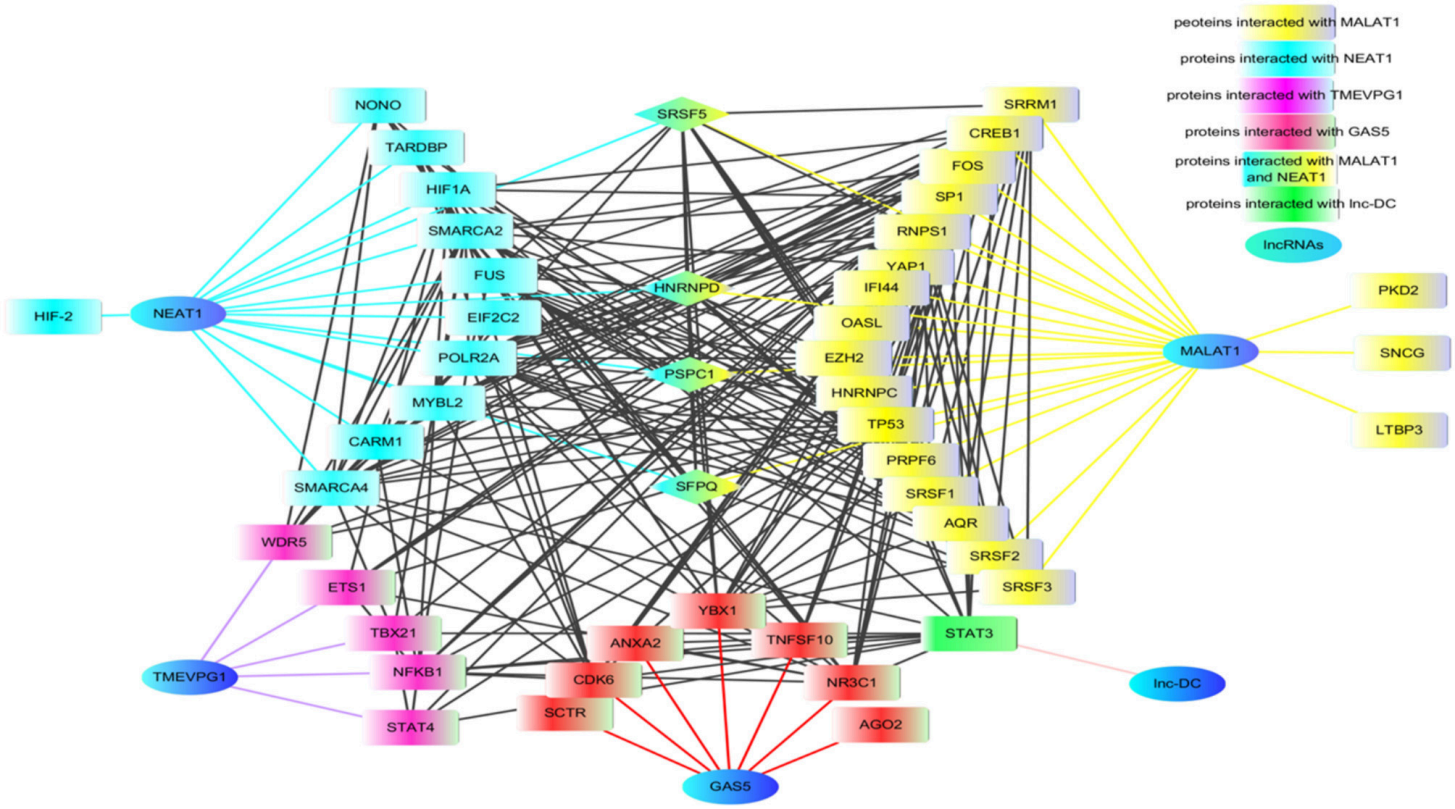
The proteins network that interacted with lncRNAs. Using lncRInter websever (http://bioinfo.life.hust.edu.cn/lncRInter/), we obtained the proteins related with lncRNAs. For those lncRNAs, did not record in the lncRInter, we got its related proteins by searching literature manually. A list of proteins interacted with lncRNAs that are implicated in RA, SLE, and pSS, respectively, were submitted to STRING website to analyze the proteins shared by multiple lncRNAs. The results from STRING were further modified by software cytoscape (version 3.6). In the network, the proteins SRSF5, HNENPD, PSPC1 and SFPQ shared by lncRNAs MALAT1 and NEAT1. The protein NONO, SFPQ, and PSPC1 are components of paraspeckle which was the key element of HDP-RNP. The color blue indicates lncRNAs, the color yellow indicates proteins related with MALAT1, the color wathet blue indicated proteins interacted with NEAT1, the color wathet blue and yellow indicates the proteins shared by MALAT1 and NEAT1, the color red indicated proteins interacted with GAS5, the color purple indicated proteins related with TMEVP1, the color green indicated proteins interacted with lnc.,-DC. SRSF5, serine and arginine rich splicing factor 5; HNENPD, heterogeneous nuclear ribonucleoprotein D; PSPC1, paraspeckle component 1; SFPQ, splicing factor proline and glutamine rich; NONO, non-POU domain-containing octamer-binding protein; HDP-RNP, HEXIM1-DNA-PK-paraspeckle components-ribonucleoprotein complex.

We speculate that lncRNAs might contribute to the pathogenesis of rheumatoid diseases through regulating those proteins. Thus, we generated an interaction network using MALAT1, NEAT1, TEMVP1, GAS5, and lnc-DC with their related proteins to demonstrate that lncRNAs might contribute to the pathogenesis of rheumatoid diseases at multiple steps which interact with each other (Figure 2).

**Figure 3:**
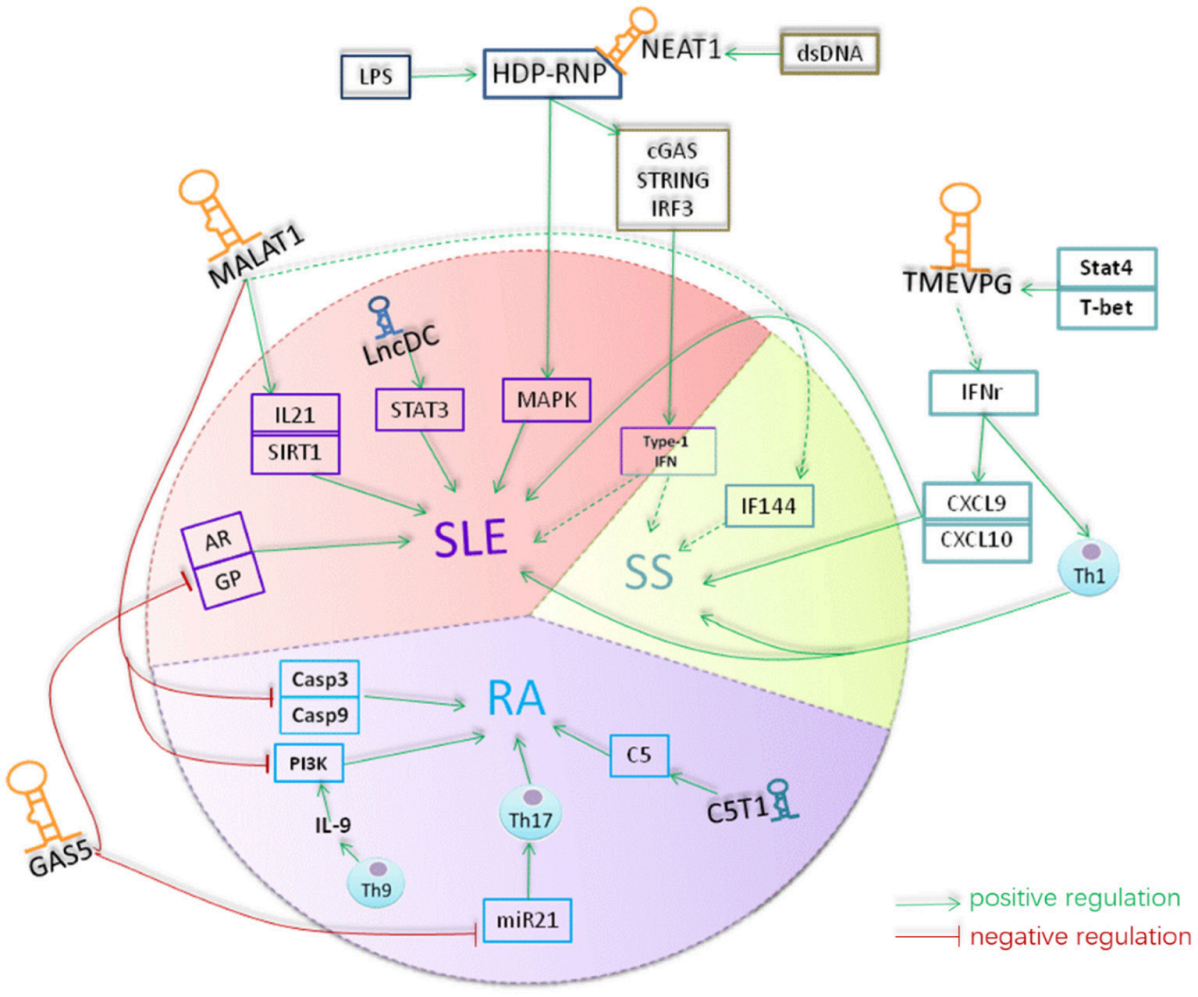
A hypothetical model for the role of lncRNAs in rheumatoid diseases. Lnc-DC was down-regulated in the plasma of SLE. Lnc-DC regulates the expression of STAT3 which plays a crucial role in Th17 differentiation. The downregulation of lnc-DC in SLE might regulate Th17 cell differentiation and thus involved in the pathogenesis of SLE. MALAT1 increases the expression of caspase3 and caspase9 and promotes the apoptosis of RAFLS cells in RA. MALAT1 is involved in the regulation of expression of E2F1 and thus the cell growth and apoptosis. Also, MALAT1 can inhibit the activity of PI3K and thus suppress the proliferation of RAFLS in RA. Th9 cells can produce IL-9 which promoting the activity of PI3K in RAFLs. The expression level of MALAT1 was increased in SLE and its expression was correlated with the expression of IL-21 and SIRT1 signaling in SLE. MALAT1 can affect the expression of IFI44 which was identified as one of the classifier genes to differentiate pSS from healthy controls. NEAT1 was up-regulated in monocyte of SLE patients with unknown mechanism. NEAT1 targeted to MAKP pathway in SLE. TMEVPG can regulate the expression of INF-γ which inducing the expression of CXCL9 and CXCL10 and thus involve in the pathogenesis of SLE and pSS. Gas5 is down-regulated in CD4 T and B cells of patients with RA and patients with SLE. mammalian target of rapamycin (mTOR)can induce the accumulation of GAS5 during growth arrest of cells. GAS5 can induce cell cycler arrest at the G0/G1 phase by inhibiting the transcription of gene such as glucocorticoid receptor and androgen. GAS5 also acted as microRNA sponge to absorb and quench microRNA like miR-21 which is essentially involved in promoting Th17 cell differentiation and contribute to pathogenesis of many autoimmune diseases. lncRNA C5T1, located in a susceptibility locus of RA, positively regulate the expression of C5 which has been suggested to play an essential role in pathogenesis of RA and its animal models. The yellow color indicates lncRNAs, the blue color indicates the molecular interacted with lncRNAs, and the red color indicated the cells that lncRNAs function. The dash arrows indicate the interaction was predicted and the solid arrows indicates the interaction were confirmed by related assay.

Based on the function and interaction of lncRNA-related proteins, here we propose a hypothetical model for the role of lncRNAs in pathogenesis of RA, SLE and SS, with a focus in TMEVPG1, MALAT1, GAS5, NEAT1, Lnc-DC, and C5T1 (Figure 3). TMEVPG1 was first identified in mice in a susceptibility locus to Theiler's virus (99). The transcription of TMEVPG1 is regulated by transcription factors STAT4 and T-bet and TMEVPG1 acting cooperator with these transcription factors positively regulates the IFN-γ expression (101). Compared with matched controls, TMEVPG1 is upregulated in the PBMC of both SS (98) and SLE (93) patients. IFNs, particularly type I IFNs, signature is a molecular feature for both in SS and SLE, which suggest that IFNs play an essential role in the two diseases (102). With regards to the IFN-γ regulated by TMEVPG1, there is an increasing body of evidence that IFN-γ plays an important role in developing of SLE and SS (103). On one hand, IFN-γ is the feature cytokine of the Th1 cells which has been demonstrated to play a role in the SLE and SS (104). On the other hand, IFN-γ could induce some chemokines, such as IFN-γ inducible 10kd protein (IP-10 or CXCL10) and CXCL9, which have important functions in attracting T cells to the target tissues in SS (105) and SLE (106). Therefore, TMEVPG1 might contribute to the development of SLE and SS through affecting Th1 cells and chemokines. In addition, since both SS (107) and SLE (108) are characterized by IFN gene signature, TMEVPG1 might also contribute to the diseases via acting on IFN-related signaling pathways.

In the patients with SLE, MALAT1 was predominantly expressed in monocytes with an abnormally increased expression level (92). MALAT1 is upregulated in FLSs of RA patients compared with corresponding controls. MALAT1 is also involved in the regulation of E2F1 expression, cell growth and apoptosis (109). In FLS from RA, the unregulated MALAT1 is associated with increased cell apoptosis, and the knockdown of MALAT1 inhibits the apoptosis of FLS by suppressing the expression of caspase-3 and caspase-9 (84), suggesting that MALAT1 contribute to the pathogenesis of RA through regulating apoptosis. In SLE, the upregulated expression of MALAT1 is positively related with SIRT1 signaling and the expression of IL-21 (92). Therefore, although MALAT1 is upregulated in both SLE and RA, it might contribute to the disease development via different mechanism. Of note, it has been shown that MALAT1 regulates interferon-induced protein 44 (IFI44) expression (110), a molecule which has been suggested to be a classifier gene to distinguish SS from healthy controls (110). Therefore, it would be interesting to evaluate the association between MALAT1 and SS. LncRNA NEAT1 colocalizes with MALAT1 to many transcription start sites. NEAT1 and MALAT1 are independent but complementary in functions (111). The expression level of NEAT1 was significantly increased in the monocytes from patients with SLE compared with controls, and the expression level of NEAT positively correlates with the clinical disease activity in SLE patient (90). NEAT1 was first identified in the mouse infected with Rabies virus (112), and the subsequent studies showed that it plays an import role in innate immune response (113). For example, NEAT1 can affect the activity of MAPK signaling thereby participates the inflammatory process mediated by TLR-4 (90). In addition, lncNRAs NEAT1 together with hexamethylene bis-acetamide-inducible protein 1 (HEXIM1), DNA-PK subunits, paraspeckle proteins and ribonucleoprotein complex can form a HEXIM1-DNA-PK-paraspeckle components-ribonucleoprotein (HDP-RNP) complex which plays key roles in DNA-mediated innate immune response through the cGAS-STING-IRF3 pathway (114). Therefore, NEAT might contribute to SS and SLE by regulating innate immune responses.

The expression of lncRNA GAS5 is significantly down regulated in both CD4+ T and B cells from RA and SLE patients compared with controls. It has been reported that GAS5 is accumulated during growth arrest induced by serum starvation or suppression of mammalian target of rapamycin (mTOR) (115). Moreover, it is known that GAS5 can induce cell cycler arrest at the G0/G1 phase by inhibiting the transcription of gene such as glucocorticoid receptor and androgen (77, 115), suggesting GAS5 might contribute to the pathogenesis of RA and SLE by regulating cell arrest. In addition, GAS5 also acted as microRNA sponge to absorb and quench microRNA like miR-21 (116). Since miR-21 is essentially involved in promoting Th17 cell differentiation and contribute to pathogenesis of many autoimmune diseases, the down regulated GAS5 might contribute to RA and SLE through uncontrolled miR-21 and Th17 cells (117) (Figure 3).

Another lncRNA of interest is lnc-DC which is exclusively expressed in DC (55). Lnc-DC is down-regulated in the plasma of SLE and associated with the presence of lupus nephritis (89). In the cytoplasm, lnc-DC directly binds to STAT3, thus promoting STAT3 phosphorylation by preventing the interaction between STAT3 and SHP1 (55). Therefore, lnc-DC might contribute to SLE development by regulating DC differentiation. It is reported that incomplete differentiation of pluripotent progenitor cells contributes to rheumatic diseases pathogenesis (118). Thus, lncRNAs which impaired the differentiation of pluripotent progenitor cells such as HOXA transcript antisense RNA, myeloid-specific 1(HOTAIRM1) (119) and H19 (120) may also contribute to the pathology of rheumatic disease.

Finally, lncRNA C5T1 has been implicated in RA because it is located in a susceptibility locus of RA (71). C5T1 positively regulate C5 expression. Complement activation has been suggested to play an essential role in pathogenesis of RA (79). Therefore, C5T1 might play a role in the pathogenesis of RA by regulating the complement system.

Those results indicate the relation of lncRNAs and rheumatic diseases pathogenesis, but further researches on animal model or clinical samples needed to confirm the role of lncRNAs in rheumatic diseases.

## Conclusions

LncRNAs plays an important role in gene regulation network during rheumatoid diseases development,thus lncRNAs may become an important target to reveal disease mechanisms and therapy. Recently, the role of lncRNAs in rheumatoid diseases has been extensively investigated. A large number of lncRNAs have been identified to be differentially expressed in rheumatoid diseases as compared with corresponding controls. However, it need to be mentioned that differential expression of lncRNAs is not by default a sign for contribution of them in rheumatic disease pathogenesis. Besides abnormal expression, more evidence are required for the identification of lncRNAs contributing to rheumatoid disease, such as association with clinical characteristics or therapeutic efficacy. Moreover, the role of lncRNA need to be validated and investigated in animal models of rheumatoid diseases.

In this review, we summarized 27, 10, and 11 lncRNAs have been implicated in RA, SLE, and pSS respectively. We discussed some possible mechanisms of these lncRNAs in detail based on published literature. By sharing lncRNAs and related proteins network analysis, we specially found that multiple components of the HDP-RNP pathway were involved in the disease, suggesting that lncRNAs regulated innate immune responses could have important roles in rheumatoid diseases.

In conclusion, accumulated evidence suggests that lncRNAs contribute to the pathogenesis of rheumatoid diseases in the past decade. Further identification of novel disease-related lncRNAs and exploration of the role of them will help us to understand the pathogenesis. We believe that the disease-related functional lncRNAs would be promising diseases biomarkers and therapeutic targets. The development of new RNA delivery entities like nanoparticles (121) and modifications such as CRISPRi (122) will promote the lncRNA-based therapeutics for a broader range of disease in the future.

## Author contributions

JZ, XY, and YG wrote the manuscript. SL prepared manuscript figure. JZ and ZZ edited the manuscript. All authors approved it for publication.

### Conflict of interest statement

The authors declare that the research was conducted in the absence of any commercial or financial relationships that could be construed as a potential conflict of interest. The reviewer SG and handling Editor declared their shared affiliation.
